# Sensitive electrochemical nonenzymatic glucose sensing based on anodized CuO nanowires on three-dimensional porous copper foam

**DOI:** 10.1038/srep16115

**Published:** 2015-11-02

**Authors:** Zhenzhen Li, Yan Chen, Yanmei Xin, Zhonghai Zhang

**Affiliations:** 1 School of Chemistry and Molecular Engineering, East China Normal University, 500 Dongchuan Road, Shanghai 200241, China

## Abstract

In this work, we proposed to utilize three-dimensional porous copper foam (CF) as conductive substrate and precursor of *in-situ* growth CuO nanowires (NWs) for fabricating electrochemical nonenzymatic glucose sensors. The CF supplied high surface area due to its unique three-dimensional porous foam structure, and thus resulted in high sensitivity for glucose detection. The CuO NWs/CF based nonenzymatic sensors presented reliable selectivity, good repeatability, reproducibility, and stability. In addition, the CuO NWs/CF based nonenzymatic sensors have been employed for practical applications, and the glucose concentration in human serum was measured to be 4.96 ± 0.06 mM, agreed well with the value measured from the commercial available glucose sensor in hospital, and the glucose concentration in saliva was also estimated to be 0.91 ± 0.04 mM, which indicated that the CuO NWs/CF owned the possibility for noninvasive glucose detection. The rational design of CuO NWs/CF provided an efficient strategy for fabricating of electrochemical nonenzymatic biosensors.

Accurate detection of glucose level in blood is of immense scientific importance for clinical diagnostics in diabetes control^1^,^2^,^3^. Traditionally, glucose concentration was monitored by enzyme-based methods with high selectivity^4^,^5^,^6^,^7^,^8^, however, which methods suffered from their intrinsic drawbacks associated with complicated enzyme purification and immobilization procedures, denatured instability, high cost, and low sensitivity due to indirect electron transfer^9^,^10^. Therefore, it is urgent to rationally design an enzyme-free method with high sensitivity and stability. Under such circumstances, electrochemical nonenzymatic method, direct electrocatalytic detecting of glucose, have received significantly attentions due to their direct-electron-transfer-shuttle-free detecting style^11^,^12^,^13^,^14^,^15^,^16^.

For successfully fabricating electrochemical nonenzymatic glucose sensor, the electrocatalyst should satisfy these requirements: (1) high electrocatalytic activity; (2) large surface area; (3) effective electron transfer from electrocatalysts to conductive substrate; (4) good selectivity; (5) high stability; and (6) good reproducibility^17^,^18^. Up to now, many nanomaterials have been employed to fabricate electrochemical nonenzymatic glucose sensor, but limited successes were achieved to satisfy all of strict requirements^19^,^20^. Generally, in a conventional fabrication process of electrochemical nonenzymatic sensor, the electrocatalysts were prepared in nanoparticles form, and then immobilized on conductive substrates with the help from certain polymer, such as Nafion, which inevitably increased the series resistance, blocked the catalytic active sites, impeded the electrolyte diffusion, ultimately leading to a significantly reduced electrocatalytic activity, poor reproducibility, and low stability^21^,^22^. Therefore, it is promising to design the electrocatalysts seamlessly connected with the conductive substrate. Among the synthesis methods, electrochemical anodization stands out owing to its versatility, one step synthesis, low-cost, and naturally seamless connection between the conductive metal substrate and the anodized metal oxides nanostructures^23^,^24^,^25^,^26^. As one of the best candidates for fabricating electrochemical nonenzymatic glucose sensor, anodized cupric oxide nanowires (CuO NWs) distinguished itself due to its superior electrocatalytic activity, and the possibility of *in-situ* direct etching from conductive copper substrate, which promoted direct electron transfer at a low potential. Some attempts have been reported to fabricate CuO nanowire or nanotubes on copper foil or sheet^27^,^28^, however, the two-dimensional surface of these substrates resulted in low catalytic active area, thus limited its sensitivity.

Herein, for circumventing this issue, we proposed to prepare one-dimensional CuO NWs from a three-dimensional porous copper foam (CF). The CF was selected as the base material due to its naturally electronic conductivity, possibility as precursor for growing CuO NWs directly, and its unique three-dimensional porous foam structure with much open area, supplied much higher surface area than Cu sheet/foil, and benefitted the flow of electrolyte^29^,^30^. All of these advantages guar-anteed both direct electron transfer and large surface area. As a proof of concept, the CuO NWs/CF was utilized for fabricating electrochemical nonenzymatic glucose sensor, and realized a sensitive glucose detecting in wide linear range with high stability, good reproducibility, and reliable selectivity.

## Results

### Preparation of CuO NWs/CF

**Figure 1:**
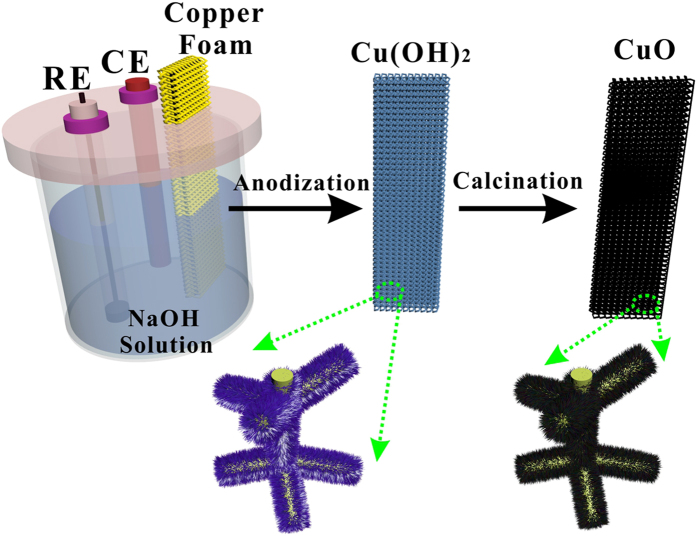
A schematic illustration of preparation of CuO NWs/CF electrode. Materials Characterization.

The design and fabrication strategy of CuO NWs on CF were presented in Fig. 1. The detailed preparation processes can be found in Methods. Briefly, the cleaned CF (coppery color, image in Supplementary Fig. S1a) was electrochemically anodized in a conventional three-electrode system to form Cu(OH)_2_ NWs (faint blue, image in Supplementary Fig. S1b) on its surface, then, the Cu(OH)_2_/CF was annealed at 180 °C for 2 h to convert Cu(OH)_2_ NWs to CuO NWs (black, image in Supplementary Fig. S1c).

**Figure 2:**
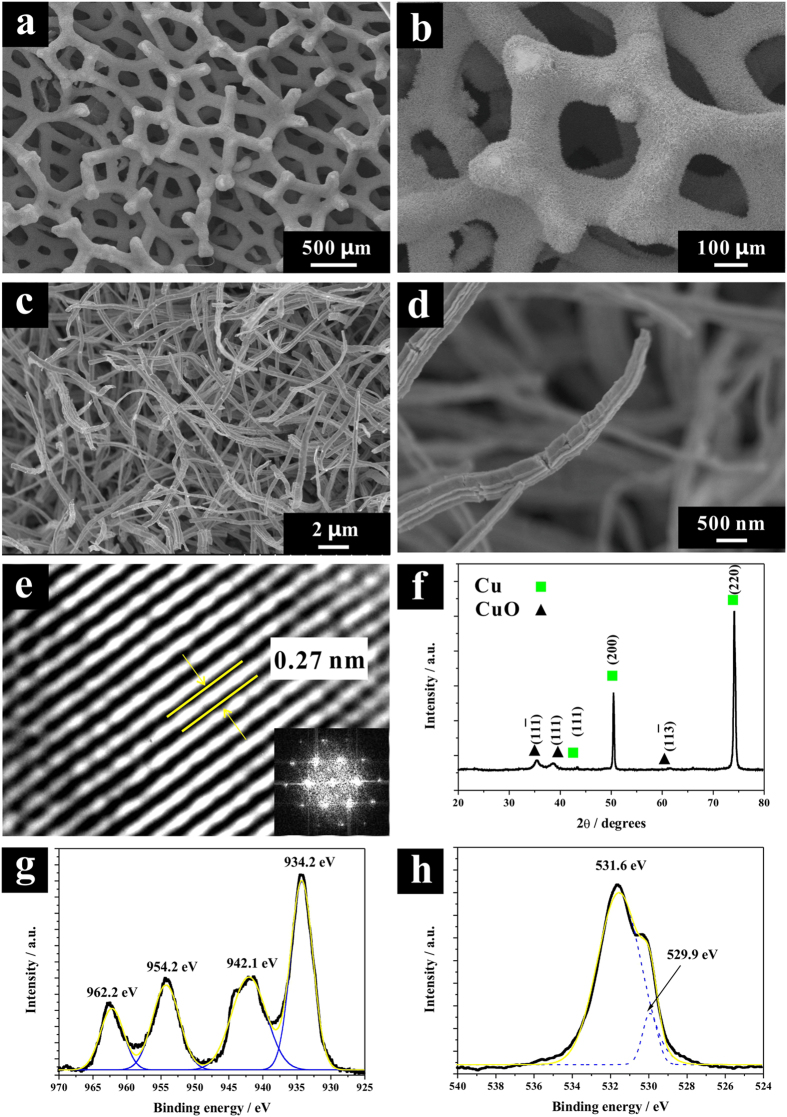
Material characterizations. (**a–d**) SEM images in various magnifications; (**e**) HRTEM image, the inset shows SAED pattern; (**f**) XRD pattern; core-level XPS of (**g**) Cu 2p and (**h**) O 1 s, of CuO NWs/CF.

Figure 2a–d presented the scanning electron microscopy (SEM) images of the CuO NWs on CF with different magnifications. As can be seen, a dense layer of the CuO NWs was uniformly covered the CF substrate, different from the Cu(OH)_2_ NWs (SEM images in Supplementary Fig. S2), the Cu NWs presented much rough surface, which would supply more active surface area for catalysis. The single CuO NWs displayed a diameter of around 300 nm, with the length of around 8~10 μm, which structure was feasible for electrolyte diffusion. From the high resolution transmission electron microscopy (HRTEM) image, as shown in Fig. 2e and Supplementary Fig. S3, the distance between two neighboring lattice fringes of CuO NWs was measured as 0.27 nm, which agreed well with the [110] lattice fringe of the monoclinic CuO^31^. Its corresponding SAED pattern (inset in Fig. 2e) helped to confirm the single crystallinity of as-grown CuO NWs.

The crystal structure of Cu(OH)_2_ NWs and CuO NWs on CF were characterized by X-ray diffraction (XRD) and presented in Supplementary Fig. S4 and Fig. 2f respectively. Both of them shown one weak diffraction peak at 43.3° and two strong diffraction peaks at 50.4° and 74.1°, which came from the CF substrate (JCPDS 04-0836). The XRD pattern of the as-anodized Cu(OH)_2_ NWs displayed pure Cu(OH)_2_ crystallinity (JCPDS 80-0656), no impurities were observed. The diffraction peaks of CuO NWs positioned at 35.5°, 38.7°, and 61.5°, can be assigned to (11-1), (111), and (11-3) planes of monoclinic CuO phase (JCPDS 48-1548). Except the characteristic CuO peaks, no peaks for other impurities or other crystal phases such as Cu_2_O and Cu(OH)_2_ were observed, which confirmed that the as–grown CuO NWs were high purity crystalline. According to the Debye-Scherrer equation^32^, the average crystal size of CuO NWs was estimated to be ~7.9 nm.

The X-ray photoelectron spectroscopy (XPS) of Cu 2p core level of CuO NWs/CF was presented in Fig. 2g, where two peaks located at 934.2 and 954.2 eV can be assigned to the binding energy of Cu 2p_3/2_ and Cu 2p_1/2_ respectively, indicating the presence of the Cu^2+^ on the sample^33^,^34^. In addition, two extra shake-up satellite peaks for Cu 2p_3/2_ and Cu 2p_1/2_ at 942.1 and 962.2 eV were also observed at higher binding energy side, implying the presence of an unfilled Cu 3d^9^ shell and thus further confirming the existence of Cu^2+^ on the sample surface. Compared with the Cu(OH)_2_ NWs sample (Supplementary Fig. S5a), the Cu 2p of CuO NWs sample presented lower binding energy, which implied that the CuO NWs owned better electron transfer ability than Cu(OH)_2_ NWs to CF substrate^35^. The core-level XPS of O 1 s of CuO NWs in Fig. 2h presented two band peaks at 529.9 and 531.6 eV, which can be ascribed to oxygen in CuO lattice and hydroxyl adsorption on its surface^36^. The core-level XPS of O 1 s of Cu(OH)_2_ NWs was also measured (Supplementary Fig. S5b) and presented one strong peak at 531.9 eV and one weak peak in 533.4 eV, which can be ascribed to hydroxyl group of Cu(OH)_2_, and chemisorbed oxygen on its surface respectively^37^.

**Figure 3:**
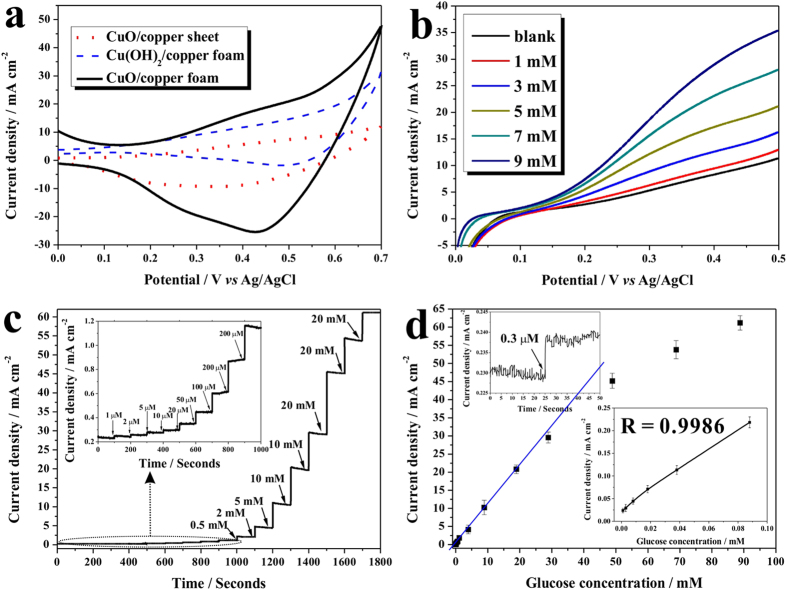
Electrochemical nonenzymatic detection. (**a**) Cyclic voltammograms of the Cu(OH)_2_ NWs/CF, CuO NWs/copper sheet, and CuO NWs/CF in 1.0 M NaOH in presence of 4 m glucose with scan rate of 50 mV s^−1^; (**b**) linear sweep voltammograms of the CuO NWs/CF electrode in various glucose concentrations with scan rate of 50 mV s^−1^; (**c**) amperometric responses of the CuO NWs/CF electrode with successive addition of glucose at 0.35 V *vs* Ag/AgCl, the inset is the enlarged amperometric responses with low glucose concentration; (**d**) the current-glucose concentration calibration curve, the right-bottom inset is the magnified calibration curve for low concentration of glucose, and the left-top inset is the current response for determining detection limit.

The electrochemical properties of Cu(OH)_2_ NWs/CF, CuO NWs/copper sheet, and CuO NWs/CF samples were investigated with a cyclic voltammetry (CV) method in 1.0 M NaOH solution with in presence of 4 mM glucose. As shown in Fig. 3a, all samples presented oxidation peak in range of 0.3–0.5 V, which can be attributed to the conversion of Cu(II) to Cu(III). Although the exact mechanism for glucose oxidation on CuO electrode in an alkaline medium was still under debate, the most accepted mechanism was first suggested by Kuwana and his coworkers^38^. Where the Cu(III) species were proposed to act as an electron-transfer medium, and the oxidative Cu(III) could catalyze glucose oxidation to generate gluconolactone and then further oxidized to glucose acid. Among all samples, the CuO NWs/CF displayed the highest anodic current density, implying its highest electrocatalytic performance. This result can be ascribed to the large surface area of three-dimensional porous structure of CF.

A series of linear-sweep voltammograms (LSV) were recorded on CuO NWs/CF at various concentrations of glucose and presented in Fig. 3b. Clearly, the CuO NWs/CF electrode did not show obvious anodic current peak in blank NaOH solution, but as increasing the glucose concentrations, the anodic current increased and strongly depended on the glucose concentrations, and all the oxidative current peaked between 0.3 to 0.4 V *vs* Ag/AgCl. Therefore, the potential of 0.35 V *vs* Ag/AgCl was selected as sensing voltage for subsequent amperometric tests to optimize the electrocatalytic response as well as obtain better sensitivity.

The CV responses of CuO NWs/CF electrode in presence of glucose at different scan rate were also recorded and presented in Supplementary Fig. S6, both anodic and cathodic peak currents varied linearly with potential scan rate in the range of 10 to 50 mV s^−1^, which suggested that the redox reaction was a surface-confined process, and the glucose molecules were direct oxidized on the surface of CuO NWs/CF electrode.

The amperometric test of the CuO NWs/CF electrode was performed and presented in Fig. 3c, where the CuO NWs/CF electrode produced an excellent amperometric response with short response time, and presented in wide response range. The inset in Fig. 3c enlarged the amperometric response of CuO NWs/CF electrode for glucose in low concentrations, which clearly showed the sensitive response for glucose on this electrode. A calibration curve was plotted and presented in Fig. 3d, which exhibited a wide response range from 1.0 μM to 18.8 mM. The plots for low concentrations of glucose was magnified and presented in the right-bottom inset in Fig. 3d, where a clearer linear relationship of glucose concentrations to current density was presented with R of 0.9986, and a high sensitivity of 2217.4 μA cm^−2^ mM^−1^ was obtained, which was not the highest value as reported for glucose sensing (Table 1), but the sensitivity and linear range were superior to most of the sensors. In addition, the detection limit was also estimated to be 0.3 μM (S/N = 3), as shown in left-top inset of Fig. 3d. The low detection limit obtained in this work allowed this sensor to be used for noninvasive detection of glucose in other biological fluids (urine, saliva, and sweat). The high sensitivity and low detection limit can be ascribed to the high surface area and direct electron transfer on CuO NWs/CF electrode.

## Discussion

**Figure 4:**
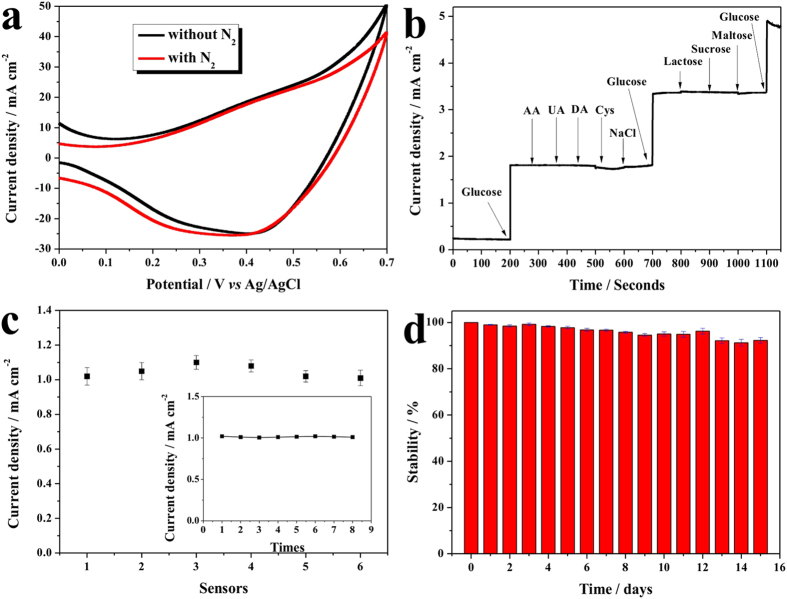
Sensors performance. (**a**) Cyclic voltammograms of CuO NWs/CF with or without nitrogen bubbling; (**b**) anti-interference property of the CuO NWs/CF electrode with initial addition of 1.0 mM glucose and 0.1 mM of AA, UA, DA, 0.5 mM cysteine, 0.1 M NaCl and then again 1.0 mM gluocse, followed by addition of 0.05 mM lactose, sucrose, and maltose, and last addition of 1.0 mM glucose; (**c**) reproducibility of six CuO NWs/CF electrodes for detection of 0.5 mM glucose, the inset is the repeatability of CuO NWs/CF electrode for detecting 0.5 mM glucose for eight times; (**d**) the stability measurement of CuO NWs/CF electrode for 15 days.

For further confirming that the enhanced current on CuO NWs/CF electrode was stemmed from the direct oxidization of glucose and studying its anti-inference activity, a series experiments were performed. First, the CVs were performed on CuO NWs/CF electrode with and without bubbling nitrogen in NaOH solution with 4.0 mM glucose, and recorded in Fig. 4a. It was observed that the glucose oxidative peak current shown insignificant change after the solution was saturated with nitrogen, which indicated that oxygen was not involved in this reaction and direct oxidation of glucose contributed to the observed current. Anti-interference properties were other essential parameters for electrochemical nonenzymatic glucose sensing, and the good selectivity would ensure a high accuracy. The selectivity of the CuO NWs/CF was tested with various potentially interfering reagents. As shown in Fig. 4b, the addition of 1.0 mM of glucose resulted in a quick and significant current increase, whereas an addition of 0.1 mM of ascorbic acid (AA), uric acid (UA), dopamine (DA), and 0.05 mM such saccharides as lactose, sucrose, and maltose did not cause observable current changes. The interference of amino acids was also studied with cysteine as a representative, which also did not cause significant current change till the concentration was up to 0.5 mM. Considering that the concentrations of these tested interfering substances in human serum are substantially lower than that of glucose^45^,^46^, the CuO NWs/CF electrode showed reliable anti-interference property and would be suitable for selective glucose detection. The favourable selectivity can be ascribed to high basic experimental pH condition, and also the high anti-interference property of CuO NWs/CF electrode. In addition, the chloride poisoning of the electrode was also checked by introducing sodium chloride (main chloride specie in human serum), which shown no significant response up to 0.1 M (similar NaCl concentration in serum), which implied that the CuO NWs/CF electrode was excellent poison resistance.

Furthermore, the reproducibility, repeatability, and stability of CuO NWs/CF electrode were also investigated. To evaluate the electrode-to-electrode reproducibility, six CuO NWs/CF electrodes were prepared under same conditions, as shown in Fig. 4c, and a good relative standard deviation of 3.51% was achieved. The repeatability of CuO NWs/CF electrode was also measured with one electrode to detect 0.5 mM glucose eight times and a relative standard deviation (RSD) of 1.57% was obtained (inset in Fig. 4c). In addition, the long-term stability of the CuO NWs/CF electrode was also tested intermittently in a period of 15 days, and a good repeatability was observed (Fig. 4d). The good reproducibility, repeatability, and stability suggested that the CuO NWs/CF electrode was quite reliable for the glucose sensing. The practical application of the CuO NWs/CF electrode was evaluated by determination of glucose in healthy adult human serum and saliva. The glucose concentration in serum was measured to be 4.96 ± 0.06 mM, which was agreed well with the value measured from the commercial available glucose sensor in hospital. The glucose concentration in saliva was also estimated to be 0.91 ± 0.04 mM mM, which indicated that the CuO NWs/CF owned the possibility for noninvasive glucose detection.

In summary, we have successfully fabricated the CuO NWs/CF electrode for highly sensitive electrochemical nonenzymatic glucose sensing, and demonstrated that the high surface area benefitted from the unique three-dimensional porous foam structure of CF, which contributed both the excellent electrocatalytic activity and high sensitivity.

## Methods

### Materials

All the chemicals were of analytical grade and used as purchased without further purification. CF (thickness of 1 mm) were supplied by Suzhou Taili New Energy Materials Ltd. Co (P.R. China). Sodium hydroxide, sodium chloride, glucose, dopamine, ascorbic acid, uric acid, cysteine, sucrose, lactose, and maltose were supplied by Macklin Inc, Shanghai, China.

### Synthesis of CuO NWs/CF electrode

The Cu foam was anodized in an alkali solution (3 M NaOH) for 30 min under 10 mA cm^−2^ to form Cu(OH)_2_ nanowire. The temperature of the electrochemical cells was maintained at 25 °C for all experiments. The as-anodized nanowire was annealed at 180 °C for 1 h to converted Cu(OH)_2_ to CuO.

### Materials Characterization

The electrodes morphologies were characterized by scanning electron microscopy (SEM, Hitachi S4800), and high resolution transmission electron microscopy (HRTEM, JEOL JEM 2100). The crystalline structure of the samples was analyzed by X-ray diffraction (XRD) (Bruker D8 Discover diffractometer, using Cu Kα radiation (1.540598 Å)). The chemical compositions and status were analyzed by X-ray Photoelectron Spectroscopy (XPS) with an Axis Ultra instrument (Kratos Analytical) under ultrahigh vacuum (<10^−8^ torr) and by using a monochromatic Al Kα X-ray source. The adventitious carbon 1 s peak was calibrated at 285.0 eV and used as an internal standard to compensate for any charging effects.

### Electrochemical nonenzymatic detection

All the electrochemical measurements including cyclic voltammetry, linear sweep voltammograms, and chronoamperometry woooooere performed with CHI 660E electrochemical working station in a three-electrode system with CuO NWs/CF as working electrode (geometrical area in solution is 1.0 cm^2^), a platinum foil as the counter electrode, and Ag/AgCl with saturated KCl solution as the reference electrode.

## Additional Information

**How to cite this article**: Li, Z. *et al.* Sensitive electrochemical nonenzymatic glucose sensing based on anodized CuO nanowires on three-dimensional porous copper foam. *Sci. Rep.* **5**, 16115; doi: 10.1038/srep16115 (2015).

## Supplementary Material

Supplementary Information

## Figures and Tables

**Table 1:** Comparison of the electrochemical nonenzymatic glucose sensor based on pristine CuO electrodes.

Electrode	Detection Potential	Sensitivity/μA cm^−2^ mM^−1^	Linear range/mM	Detection limit/μM	Reference
CuO nanowires/ copper foam	0.35 V *vs* Ag/AgCl	2217.4	0.001–18.8	0.3	This work
CuO nanotubes/copper foil	0.32 V *vs* SCE	1890	0.005–3	0.1	25
CuO nanourchins	0.50 V *vs* Ag/AgCl	2682	0.1–3	1.52	21
inkjet printed CuO nanoparticles	0.60 V *vs* Ag/AgCl	2762.5	0.05–18.45	0.5	31
CuO nanospheres	0.60 V *vs* Ag/AgCl	404.53	0.05–2.55	1	39
CuO nanoparticles	0.40 V *vs* Ag/AgCl	1430	0.04–6	5	40
CuO nanowires	0.55 V *vs* Ag/AgCl	648.2	NA	2	41
Cu nanowires/Cu	0.33 V *vs* Ag/AgCl	490	0.0004–2	0.049	42
CuO nanfibers	0.40 V *vs* SCE	431.3	0.006–2.5	0.8	43
CuO nanoflowers	0.50 V *vs* Ag/AgCl	2657	0.01–5	1.71	44
