# Supplementary material for: Sensitive electrochemical nonenzymatic glucose sensing based on anodized CuO nanowires on three-dimensional porous copper foam
Source: Sci Rep. 2015 Nov 2;5:16115. doi: 10.1038/srep16115 (PMC4629205; doi:10.1038/srep16115)
Supplement: Supplementary Information [file srep16115-s1.pdf]

Supplementary

Sensitive electrochemical nonenzymatic glucose sensing based on anodized CuO nanowires on three-dimensional porous copper foam

Zhenzhen Li, Yan Chen, Yanmei Xin, Zhonghai Zhang*

School of Chemistry and Molecular Engineering, East China Normal University, 500 Dongchuan Road, Shanghai 200241, China.

Email: zhzhang@chem.ecnu.edu.cn

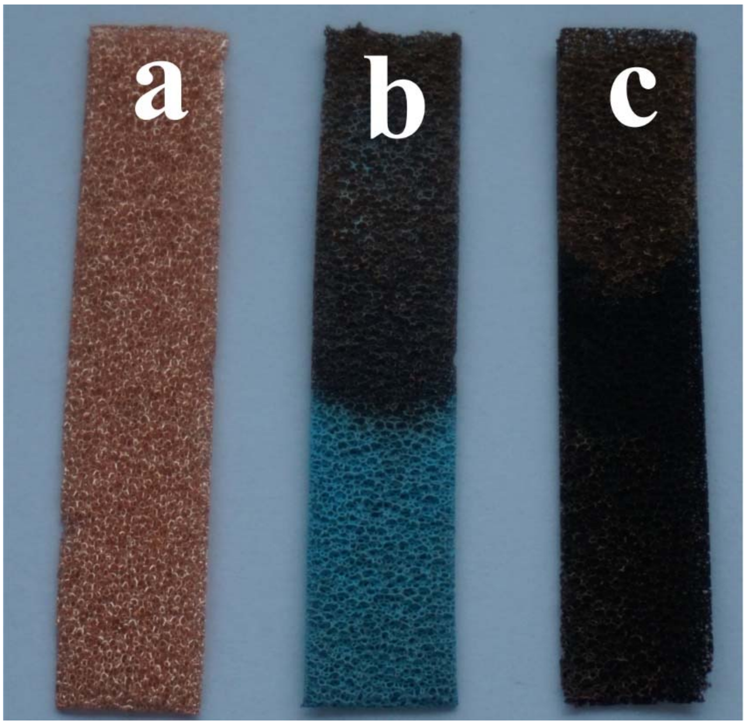

Figure S1 | Digital photos of (a) CF, (b) $Cu(OH)_2$/CF, and CuO/CF.

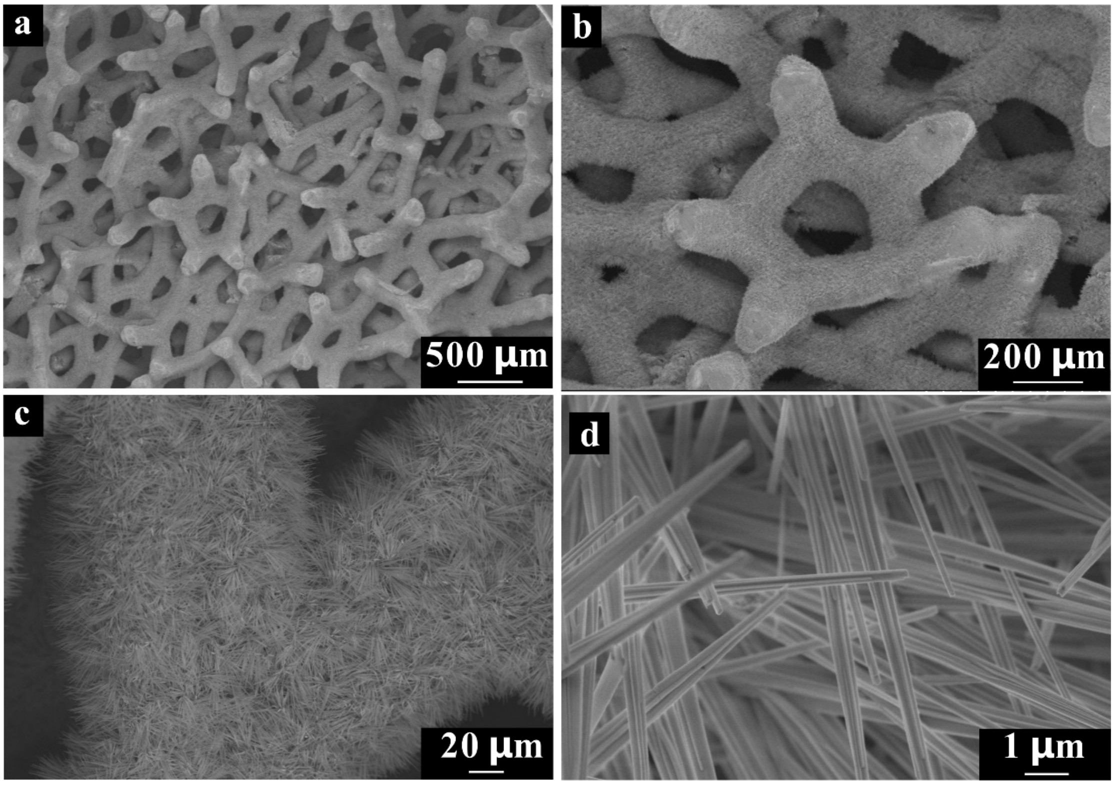

Figure S2 │ SEM images of $Cu(OH)_2$ NWs/CF with different magnifications.

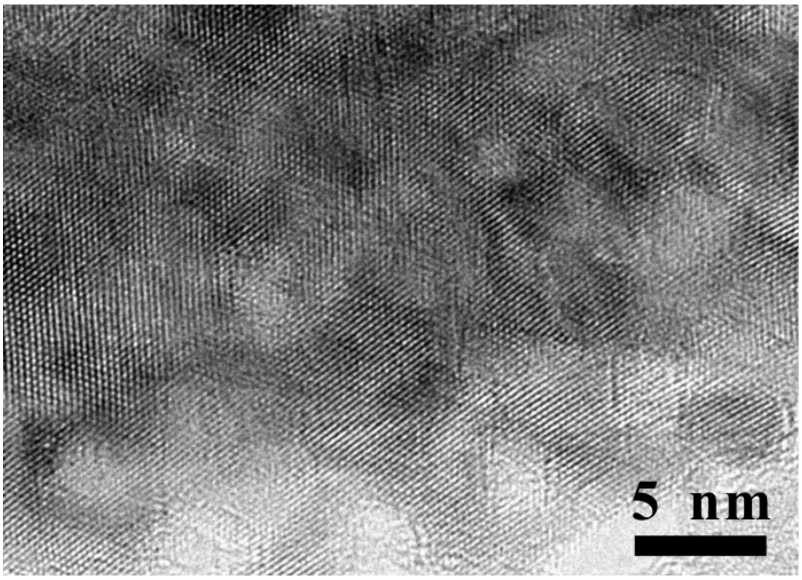

Figure S3 │ HRTEM of CuO NWs/CF.

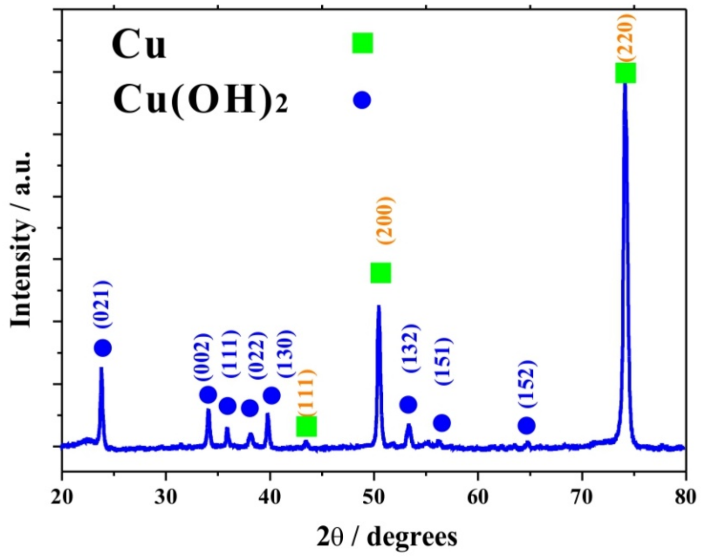

Figure S4 │ XRD pattern of $Cu(OH)_2$ NWs/CF.

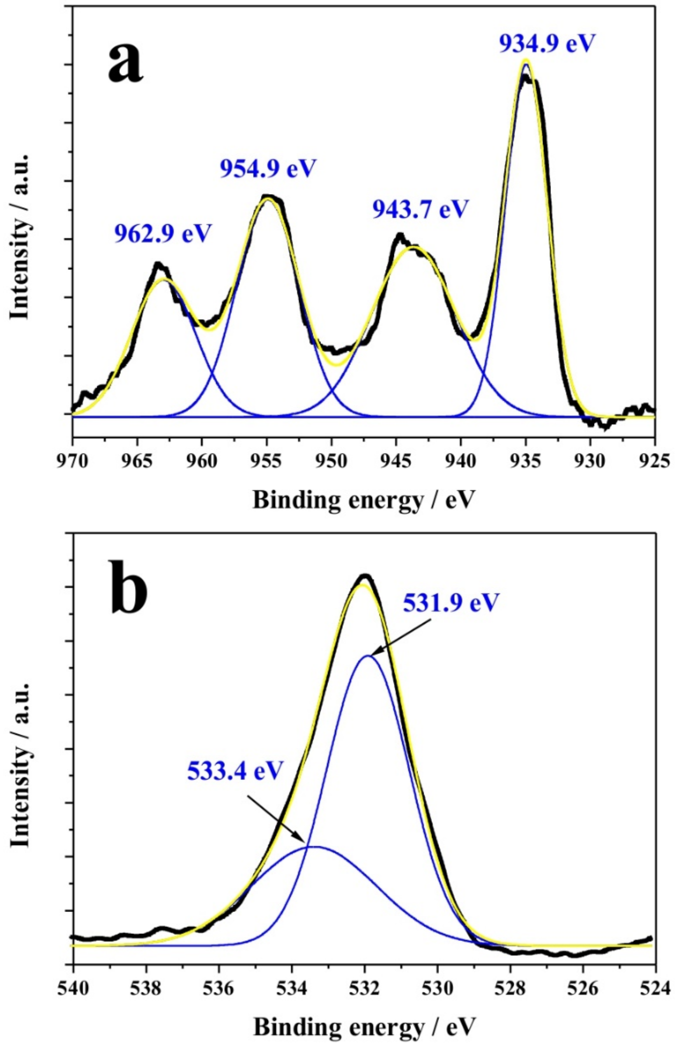

Figure S5 │ Core-level XPS of (a) Cu 2p and (b) O 1s of $Cu(OH)_2$ NWs/CF.

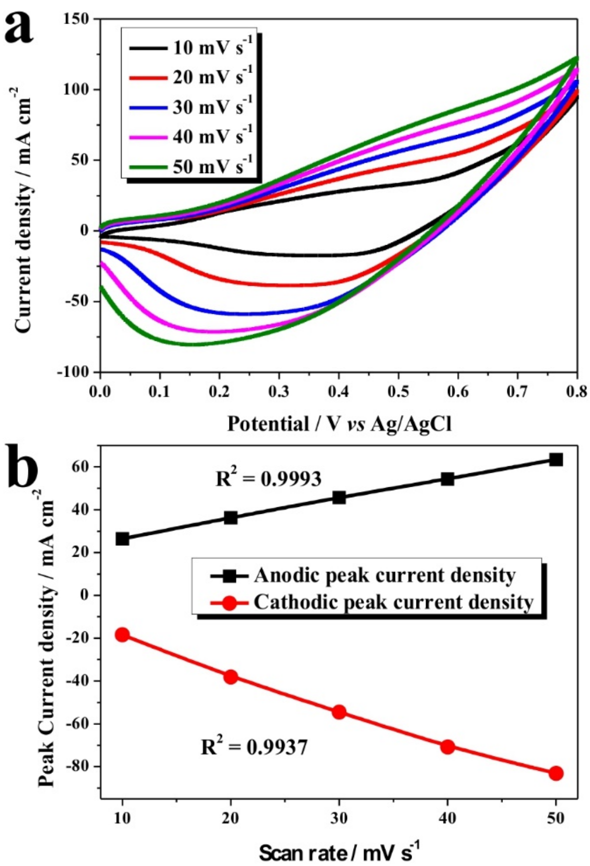

Figure S6 │ Cyclic voltammograms obtained on CuO NWs/CF electrode in presence of 4 mM glucose in 1.0 M NaOH at different scan rates, (b) the relationship between peak current with scan rates.
